# 

**Published:** 1875-12

**Authors:** 


### INDEX.

A — PAGE

A Deserved Compliment .............. 478
Abdominal Wound caused by a Bull's Horn—W. A. Carmichael, M.D.... 733
Acute Articular Rheumatism, Treatment of, with Starch Bandage ..... 927
Adolphus, Philip, M.D., The Obstetric Forceps ........................ 130
Aerotherapy in Chlorosis................ 907
Albany Medical College—Meeting of the Alumni of ...................... 157
Alcohol, Its Therapeutic Action and Remedial Agency—J. S. Whitmire, M.D .................................. 172
Allen, J. Adams, M.D., Medical News and Gossip.......................... 617
Amputation through Joints.......... 685
Amputations, Statistics of............ 763
Analogies of Dislocation of Shoulder and Hip Joints.. .................... 921
Andrews, E., M.D., Cinchonidia, Sulphate of.............................. 649
Anal Eczema ... ........................ 682
Announcements for the Month of September, 1875......................... 720
Announcements for the Month of October, 1875............................ 800
Announcements for the Month of November, 1875......................... 880
Announcements for the Month of December, 1875...................... 960
Antiseptic Treatment at Halle, Germany 763
Apocynum Cannabinum in Dropsical Affections.......................... 772
Aphasia, two Cases of—Walter Hay, M.D ........... ................. 561
Aphasia, Historical Notice concerning, 956

B

Bandages, Plaster Paris................ 848
Battey's Operation ..................... 912
Benedict, Dr. Moriz, Variations in the Pulse of Different Arteries......... 706
Bills of Interest to the Medical Profession .................................... 310
Blaney, J. V. Z., Death of... ........... 3
Bladder, Stone in the—E. O. Grattan, M.D................................ 569
Bladder, Extirpation of a Tumor of... 761
Bladder, Female Urinary, Method of Rendering Accessible............. 834
Blood, Putrid, on the Toxic Principle of 765
Boils, Sulphuric Acid in Treatment of. 849
Book Notices—
Chicago Relief and Aid Society, Report of.............................. 57
Hempel, Science of Homœopathy... 59
Ziemssen, Dr. H. Von, Cyclopædia of Practical Medicine... .......... 61
Stille, Therapeutics and Materia Medica ............................ 154
Biddle, Materia Medica for use of Students ............................ 219
Aitken, Outlines of the Science and Practice of Medicine.... .......... 220

Book Notices— PAGE

Thompson, Free Phosphorus in Medicine ................................ 222
Woodworth, Nomenclature of Disease ................................... 225
Cohen, Croup in its Relations to Tracheotomy ....................... 226
Howe, The Breath and the Diseases which give it a Fetid Odor........ 227
Fox, The Diseases of the Stomach.. 228
Physician's Case Record............. 300
Ford's Questions in Anatomy....... 301
Transactions of the Pathological Society, Philadelphia.................. 301
Tyson, Practical Examination of Urine.................................. 302
Thompson, Clinical Lectures on Diseases of the Urinary Organs....... 303
Fowler, Examination of Urine...... 305
Gorton, The Drift of Mental Philosophy .................................. 305
Buhl, Inflammation of the Lungs... 305
Edmunds, The Medical Uses of Alcohol ................................ 306
Salter, Dental Pathology and Surgery, 393
Hartshorne's Essentials of the Principles and Practice of Medicine..... 394
Ziemssen, Cyclopædia of Practical Medicine ........................... 460
Murchison, Functional Derangements of the Liver,..................... 468
Transactions of the 24th Anniversary Meeting Illinois State Medical Society ........ ....... ............. 470
Clay, Complete Hand Book of Obstetric Surgery...................... 472
Comegys, C. G., M.D., Annual Address of............................. 606
Wagner, C., M.D., Certain Nervous Affections of the Throat.......... 608
Beard, Geo. M., A.M., M.D., A Practical Treatise on the Medical and Surgical uses of Electricity........ 608
Anderson, A Practical Treatise on Eczema.............................. 609
Knight, Jas., M.D., Orthopædia..... 610
Corson, Treatment of Pleurisy...... 610
Chambers, A Manual of Diet........ 611
Griffiths, Lessons on Prescriptions and the Art of Prescribing........ 715
Bastian, On Paralysis from Brain Disease............................. 778
Paget, Clinical Lectures and Essays, 782
Leonard, The Multum in Parvo Reference and Dose Book............. 782
Engelmann, The Mucous Membrane of the Uterus. .................... 860
Fothergill, The Heart and its Diseases ............................. 938
Fenner, C. S., M.D., Vision: its Optical Defects and the Adaptation of Spectacles ......................... 942
Baudery, J. K., M.D., Lectures on Diseases of the Nervous System... 944
Taylor, On Poisons in Relation to Med. Jurisprudence and Medicine. 946

Book Notices— PAGE
Bergmann, Results of Excisions of Joints during Franco-German War, 946
Bowel Affections of Young Children, —N. S. Davis, M.D ................ 721
Bowel Affections of Young Children—S. B. Hawley, M.D................ 881
Brain, Examination of—Jos. G. Richardson, M.D.................. 401
Bridge, Norman, M.D., Report on Practical Medicine.................. 189
Bridge, Norman, M.D., Case of Sudden Death .......................... 276
Bridge, Norman, M.D., Case of Intestinal Hæmorrhage in an Infant One Day Old.......................... 487
Bromide of Potassium, Influence of, on the Menstruation ............ 825
Bromhydrate of Quinine ... ........... 931
Bull's Horn, Abdominal Wound Caused by—W. A. Carmichael, M.D. 733

Cancer, Best Treatment of — J. E. Nichols, M.D ........................ 179
Cancer of the Pylorus—J. H. Etheridge, M.D ........................ 489
Cantharidal Strangury................ 931
Cardiac Palpitations, Arrest of......... 859
Carmichael, W. A., M. D., Extensive Abdominal Wound caused by a Bull's Horn.......................... 733
Case in Practice—B. F. Farley, M.D .. 485
Case of Hiccough successfully treated by Inhalation of Amyl Nitrite—C. Paul Simon, M.D.................. 886
Cases—R. L. Rea, M. D................ 495
Cerebral Meningitis—Walter Hay, M.D. 481
Cerebral Rheumatism.................. 924
Changes Produced by Circulation in Volume of Members................ 937
Chicago Medical Society, 130, 577, 742, 892, 898, 900
Chicago Society of Physicians and Surgeons ................44, 45, 202, 282, 368, 511, 575, 667, 670, 747, 815, 820, 901.
Chicago Mortality Report—
By Dr. Ben. C. Miller.
For November, 1874 .................. 78
" December, 1874.................. 158
" The Year 1874.................. 160
" January, 1875 .................. 238
" February, 1875 .................. 318
" March, 1875...................... 398
" April, 1875 ...................... 479
" May, 1875 ...................... 559
" June, 1875 ...................... 639
" July, 1875....................... 717
" August, 1875..................... 798
" September, 1875 ................. 878
Children, Feeble Minded .............. 235
Children, Bowel Affections of—Dr. N. S. Davis .......................... 721
Chloral, Hydrate of, in Obstetric Practice............................ 754
Chloral vs. Strychnia Poisoning—Another Case........................ 776
Still Another .. ...................... 777
Chloral in Natural Labor .............. 827
Chloride of Zinc, Various Uses of—J. E. Nichols, M.D.................... 321
Cholera, On Pathology and Treatment, 690
Cinchona Alkaloids, the Cheaper—J. S. Whitmire, M.D .................. 564
Cinchonidia, Sulphate of — Edmund Andrews, M.D...................... 649

Clinics— PAGE
Cook County Hospital ............... 54
Illinois Charitable Eye and Ear Infirmary—E. L. Holmes, M.D ....... 148
Rush Medical College—Prof. Moses Gunn, M.D ....................... 152
Illinois Charitable Eye and Ear Infirmary—E. L. Holmes, M. D....... 427
Cloth Tents—C. Henri Leonard, M.D .. 729
Cohen, L. H., M.D., Correspondence... 813
Cold Injections......................... 932
Concours, at Rush Medical College ... 72
Conjunctivitis, Acute, Tannic Acid for. 757
Conjunctiva, Transplantation of ...... 758
Convulsions due to Morbid Conditions of Non-Puerperal Uterus .......... 907
Correspondence—
L. H. Cohen, M.D..................... 813
Frank H. Hamilton, M.D............. 814
Edmund J. Doering, M.D. ........... 889
Cushing, Jno. R., M.D., Treatment of Dysentery with Creasote........... 28
Cutaneous Diseases, Local Treatment of 842

D

Danforth, I. N., M.D., Pathology and Microscopy—Cancer of the Pylorus 492
Danforth, I. N., M.D., the Microscope in Daily Practice ................... 659
Danforth, I. N., M.D., the Microscope in Daily Practice.................... 737
Danforth, I. N., M.D., The Microscope in Daily Practice.................... 807
Davis, N. S., M.D., on Bowel Affections of Young Children ................ 721
Death, Case of Sudden — Norman Bridge, M.D ........................ 276
Diabetes, Glycerine in Treatment of, 663
Diarrhœa of Typhoid Fever, Therapeutics of the ........................ 771
Diphtheritic Sore Throat, Quinine as a Gargle in .......................... 858
Dislocation of End of Clavicle upon Acromion of Scapula............... 920
Disturbance of Nutrition after Lesion of Nerves of Leg ................... 917
Douglas's Cul-de-sac, Drainage of, in Ovariotomy... ...................... 913
Dysentery, Treatment with Creasote—J. R. Cushing, M.D................ 28
Dysentery, Acute, Natural History of.. 689

E

Early Menstruation.... ................ 911
Eczema, Anal ......................... 682
Editorial—
Dr. Hay................................ 552
Valedictory ........................... 623
Personals ............................. 624
Salutatory . .......................... 664
Homœopathic College of University of Michigan ........................ 888
Electrolysis applied to Treatment of Various Tumors.................... 933
Elephantiasis Arabum................. 839
Epithelioma of the Prepuce, and a Singular Anatomical Vagary—A. L. Wright, M.D..................... 653
Erysipelas Treated by Hypodermic Injections of Carbolic Acid........ 686
Ether vs. Chloroform—E. W. Lee, M.D., 108
Etheridge, Jas. H., M.D., Cancer of the Pylorus............................ 489
Extensive Abdominal Wound, etc.—H. R. Rogers, M.D..................... 884

PAGE
Extra-Uterine Pregnancy............677, 678
Eye, Clinical Lectures on Diseases of —E. L. Holmes, M. D.............. 148

F

Farley, B. F., M.D., Case in Practice .. 485
Feeble Minded Children................ 235
Feeble Minded, Institution for......... 476
Femoral Hernia—Jno. McLean, M.D... 488
Female Urinary Bladder, Methods of Rendering Accessible.............. 834
Fevers, Intermittent, Non-malarious Origin of .......................... 852
Fingers, Webbed, Elastic Ligature for Cure of.............................. 759
Fractures, Ununited, Treatment of .... 848
Freer, Dr., Transfusion.... .. ....... 518
Function of Levator Ani Muscle...... 934
Fusiform Aneurism of the Anterior and Posterior Tibial Arteries..... 846

G

Gastro-Elytrotomy..................... 755
Gastro-Pulmonary Fistula consecutive to Gastric Ulcer..................... 929
Glycerine in Treatment of Diabetes... 663
Goa Powder and Poh di Bahia in Eczema Marginatum .............. 842
Goitre, Exophthalmic, Observations and Treatment.. ................. 854
Gratton, E. O., M.D., Stone in the Bladder............................ 569
Grave's Disease........................ 758
Gunn, Moses, M.D., Clinic by ........ 152
Gunn, Moses, M.D., Traumatic Tetanus ...................................... 421

H

Hæmorrhage in Infant One Day Old—Norman Bridge, M.D. ............ 487
Hæmorrhage, Infantile—H. M. Lyman, M.D .............................. 801
Hæmaturia, Interesting Case .......... 856
Hamilton, Frank H., on the Use of Warm and Hot Water in Surgery .. 602
Hamilton, Frank H., Correspondence.. 814
Hay, Walter, M.D., Cerebral Meningitis 481
Hay, Walter, M.D., Editorial .......... 552
Hay, Walter, M.D., Two Cases of Aphasia .............................. 561
Hawley, S. B., M.D., Bowel Affections of Young Children................ 881
Headache, Treatment of Different Forms of ... ... .. ............... 923
Hereditary Transmission and Variation by Descent ........................ 403
Hernia, Strangulated, Reduced by Taxis through Colon ............. 759
Hernia, Strangulated, Treatment of, by Subcutaneous Dilation ...... ... 760
Hernia, Femoral—Jno. McLean, M.D., 488
Hermaphrodism, from a Medico-Legal Point of View—E. Warren Sawyer, M.D...............695, 784, 867, 947
Hæmorrhagic Cerebral Tumor....... . 749
Hoadley, A. E., M.D., Case of Double Vagina and Uterus................. 735
Holmes, E. L., M.D., Illinois Charitable Eye and Ear Infirmary..... ..... 289
Holmes, E. L., M.D., Clincal Lectures on Diseases of the Eye ............ 148
Holmes, E. L., M.D., Illinois Charitable Eye and Ear Infirmary ........ 427
Hospitals—
St. Luke's ............................ 48

Hospitals— PAGE
Illinois Charitable Eye and Ear Infirmary ... ........................ 77
St. Joseph's Hospital ................. 421
Cook County Hospital.... ....54, 674, 826
Mercy Hospital ........................ 905
Hydatid Cyst in Abdomen........... ... 918
Hydatid Disease of Liver, Treatment of, 917
Hydrate of Chloral in Obstetric Practice ............................ 754
Hyperphyrexia in Typhoid Fever, Use of Electricity for........... ...... 933
Hypodermic Syringe.................... 631
Hystero-Epilepsy relieved by Pressure of Ovaries ....................... 924
Hystero-Epilepsy with Anuria......... 925
Hysteria in a Male Cured by Compression of Testicles...... . .. .. 924

I

Illinois Charitable Eye and Ear Infirmary. ..... ..................77, 289, 427
Incarceration of Jejunum and Intussusception .......... .............. 691
Infantile Hæmorrhage—H. M. Lyman, M.D..... .. ...... ............ 801
Infectious and Contagious Diseases.... 857
Intermittent Fevers, Non-malarious Origin of ........................ 852
Intestinal Hæmorrhage in an Infant one day old—Norman Bridge, M.D...... 487
Intestinal Invagination, Differential Diagnosis of...... ..................... 763
Intestinal Diseases treated by introducing large quantities of Fluid.. 765
Intussusception—a good way to distend the lower bowel in ................ 771

J

Jaborandi, Mechanism of the Action of 773
Jackson, A. Reeves, M.D., Progress in Obstetrics and Gynecology .... .. 31

K

Kidney, Gangrene of the Left.......... 853
Koumiss, as a Remedial Agent- R. L. Leonard, M.D..... .............. .... 326

L

Larynx, Foreign Body in ... ....... ... 849
Lee, E. W., M.D., Ether vs. Chloroform 108
Leonard, C. Henri, M.D., Warm and Hot Water in Surgery.................. 645
Leonard, C. Henri, M.D., Cloth Tents.. 729
Leonard, R. L., M.D., Koumiss as a Remedial Agent..... ............... 326
Loot ........................................ 627
Lyman, H. M., M.D., Infantile Hæmorrhage ........................... 801

M

Mammary Ducts, Epithelium of the.... 679
McClelland, M. A., M.D., The Medical Expert ................ 9, 81, 161, 241
McLean, Jno., M.D., Femoral Hernia.. 488
McElroy, Z. C., M.D., Syphilis: its Materials and Forces.......... ... 251
Medical Education and Medical Legislation .... ......................... 312
Medical News.............................. 521
Medical News and Gossip, by L'Ancien Editeur......... ............ .... 617
Medical Sciences, Summary of Progress in the..................676, 749, 827, 907

PAGE
Medical News and Items.......792, 876, 957
Menstruation, Vicarious................ 756
Menstruation, Influence of Bromide of Potassium on..................... ... 825
Metrorrhagia, Use of Heat in........ .. 911
Microscope in Daily Practice—I. N. Danforth, M.D..................... 659
Microscope in Daily Practice, 2d paper—I. N. Danforth, M.D................ 737
Microscope in Daily Practice, 3d Paper —I. N. Danforth, M.D.............. 807
Minor Operations in Surgery—J. L. White, M.D... ... ....... ........ 141
Movements of the Iris. .............. 915
Multiple Cutaneous Sarcomata ........ 842

N

Nævi Successfully Treated with Electrolysis ....... ..................... 685
Narcotism by Products of Tissue Change after Fatigue .............. 935
Nasal Cavities, Plugging the........... 757
Nervous System and Skin Diseases.... 684
Nervous System, Influence of, upon Phenomena of Pregnancy and Parturition................................. 751
Neuralgia, Treatment of........... ... 777
Neuralgia, Facial, Gelseminum Sempervirens in........................ 859
New Test for Amyloid Tissues ... .... 935
New Process for Staining Tissues...... 936
Nichols, J. E., M.D., Best Treatment of Cancer. .......................... 179
Nichols, J. E., M.D., Various Uses of Chloride of Zinc.................. .. 321
Non-Specific Rupia Prominens.. ..... 841

O

Obituary—
Blaney, J. V. Z., M.D................ 2
Joshua Riley, M.D. .................. 317
Mrs. Dr. Wm. J. Maynard........... 729
Headland, Dr. F. W.................. 957
Bennett, Dr. John Hughes........... 957
Obstetric Forceps—Philip Adolphus, M.D. ....... ........................ 130
Obstetrics, Progress of—E. W. Sawyer, M.D. ......... ........126, 194, 330, 500
Obstetrics.................676, 749, 827, 907
Odontalgia, Gelseminum in............ 859
Opium for Diabetes... .......... .. 926
Orbit, Case of Foreign Body in—M. G. Sloan, M.D... ..................... 497
Ovariotomy at Thirteen............. ... 912
Ovariotomy Complicating Pregnancy .. 681
Ovary, the Development of the........ 679
Owens, Jno. E., Progress of Surgery, 37, 119, 197, 279, 412, 570

P

Philadelphia, Board of Health of .... 752
Placenta Praevia, Diagnosis and Treatment of............ ............. 676
Pneumonia, Some of the Conditions under which it proves fatal ........ 768
Poisoning by Strawberries............. 673
Practice, Case in—B. F. Farley, M.D... 485
Pregnancy, Vomiting in....... . .... 627
Prepuce, Epithelioma of the... . .... 653
Pregnancy, Ovariotomy Complicating.. 681
Procedure in Right Occipito-posterior Position ............................. 755
Progress of Obstetrics—E. Warren Sawyer, M.D..... ........126, 194, 330, 500
Progress in Medical Sciences, 31, 119, 189, 279, 330, 412, 500, 570
Progress of Surgery, PAGE 37, 119, 197, 279, 412, 570
Psoriasis, Tar in.... .................. 840
Puerperal Blindness.................... 915
Pulse of Different Arteries, Variations in the.................................. 706
Purgatives ............................ 931
Pylorus, Cancer of—J. H. Etheridge, M.D......... ........................ 489

Q

Quinine as a Gargle in Diphtheritic Sore Throat................................ 858

R

Rea, R. L., M.D., Cases................ 495
Reduction of Retroverted Uterus...... 909
Reports of Societies. See "Societies."
Researches on Absorption and Elimination of Iodine ...... .... ........ 929
Respiratory Percussion................ 687
Respiration and Circulation, Diseases of, Treated by the Pneumatic Method .... ................ ....... 767
Richardson, Jos. G., M.D., Examination of the Brain.................... 401
Rogers, H. R., M.D., Extensive Abdominal Wound, etc..................... 884
Rupture of External Iliac Artery..... 845
Rupture of Ovarian Cysts into the Peritoneum ....... ................ 914
Rush Medical College, Annual Commencement.......................... 232
Rush Medical College Building, laying of Corner Stone...................... 958

S

Sawyer, E. Warren, M.D., an Instance of Blood-Losing by Venesection .. 641
Sawyer, E. Warren, M.D., Hermaphrodism...... ..........695, 784, 867, 947
Sawyer, E. Warren, M.D., Progress of Obstetrics... .........126, 194, 330, 500
Scarless Eradication of Nævi.......... 685
Sea Sickness, its Pathology and Treatment with Amyl Nitrite ........... 857
Secondary Perineal Traumatism....... 911
Selections—
On the Alleged Successful Treatment of Typhoid Fever by "Cold," 294
On the Physiology of the act of Vomiting ....................... 298
Impressions of American Surgery... 340
Case of Aneurism of the Abdominal Aorta...... ..................... 353
A Strychnia Eater .. ............... 354
Spinal Arthropathies.. .............. 355
On the Similarity between Red Blood Corpuscles of Man and those of certain other Mammals, especially the Dog.......................... 376
Explanatory Note in Regard to the Diagnosis of Blood Stains......... 390
The Neurotic Origin of Disease..431, 525
The Medical Year 1874.............. 205
On the Use of Warm and Hot Water in Surgery—F. H. Hamilton, M.D... 602
Severe Prolapsus Recti.... ............ 919
Simon, C. Paul, M.D., Case of Hiccough successfully treated by Inhalation of Amyl Nitrite..................... 886
Skin-Grafting........................... 916
Sloan, M. G., M.D., Case of Foreign Body in Orbit..... ............ .. 497

Societies, Reports of— PAGE
Chicago Medical Society, 130, 577, 742, 892, 898, 900
Central Illinois Medical Society..... 141
Chicago Society of Physicians and Surgeons..44, 45, 202, 282, 368, 511, 518, 575, 667, 670, 747, 815, 820, 901
Illinois State Medical Society.397, 503, 636
Medical Association, Alabama.... .. 558
Wisconsin State Medical Society.... 587
The Cass Co. (Indiana) Medical Society....... ........................ 596
The American Neurological Association.. .............................. 598
International Congress of Ophthalmology.. ..... ..................... 638
Rock River Medical Society......... 824
Stone in Bladder—E. O. Grattan, M.D., 569
Strawberries, Poisoning by............ 673
Sulphate of Quinia.................... 676
Sulphuric Acid in the Treatment of Boils................................ 849
Summary of Progress in the Medical Sciences..... .........676, 749, 827, 907
Surgery, Minor Operations in—J. L. White, M.D.......................... 141
Surgery, Use of Warm and Hot Water in . .............................. 602
Surgery, Warm and Hot Water in—C. Henri Leonard, M.D............... 645
Syphilis: its Materials and Forces—Z. C. McElroy, M.D................... 251
Syphilis, Preventive Measures in...... 630
Syringe, Hypodermic.................. 631
Syphilis, Visceral.................... 681

T

Tannic Acid for Acute Conjunctivitis.. 757
Tar in Psoriasis ..................... 840
Tents, Cloth—C. Henri Leonard, M.D.. 729
Tetanus, Traumatic—Moses Gunn, M.D. 421
Tinnitus Aurium ...................... 850
Trichinosis in Dearborn Co., Ind....... 770
Trismus Nascentium, Therapeutics of.. 692
Transfusion—Dr. Freer................ 518
Transfusion, Experiments of Prof. Ponfick on a Dog.................. .. 762
Traumatic Tetanus, Moses Gunn, M.D... 421
Treatment of Cephalohæmatoma by Aspiration.... ..................... 928
Treatment of Amblyopia Potatorium.. 916

PAGE
Typhoid Fever, Treatment of, by "Cold" .......................... 294
Typhoid Fever, Therapeutics of Diarrhœa in......................... 771

U

Urethra, Female, Simon's Method of Dilating .......................... 760
Uterine Polypus, Convulsions due to .. 908
Urine, Retention of, Treated by Aspiration....... ...................... 761
Uterine Spasm, Treatment of.......... 677
Uterus, Extirpation of, by Abdominal Section ..... ........... ............ 909

V

Vagina and Uterus, Case of Double—A. E. Hoadley, M.D.................. 735
Valedictory—Walter Hay, M.D......... 623
Varix ............................... 922
Varix, Treatment of.................. 847
Varicose Veins ..... ................. 686
Vesical Ecchymoses in the Newly Born ..... ........................ 751
Venesection, an Instance of Blood-Losing by—E. Warren Sawyer, M.D. 641
Vicarious Menstruation................ 756
Vocal Fremitus in Pleurisy and Pneumonia.......... ................. 631
Vomiting in Pregnancy................ 627

W

Ware, Dr. Lyman, on London Hospitals....... ......................... 717
White, J. L., M.D., Minor Operations in Surgery......................... 141
Whitmire, Jas. S., M.D., Alcohol: its Therapeutic Action and Remedial Agency ... ....................... 172
Whitmire, Jas. S., M.D., the Cheaper Cinchona Alkaloids ................ 564
Wright, A. L., M.D., Epithelioma of the Prepuce......................... 653

Y

Yellow Fever Poison, Modus Operandi of.......................... ........ 853

### ANNOUNCEMENTS FOR THE MONTH.

### MONDAYS.

SOCIETIES.

*Mondays, Dec.* 6 and 20—Chicago Med. Society, regular meetings at Gault House, 8 P.M.

*Mondays, Dec.* 13 and 27 — Chicago Society of Physicians and Surgeons, regular meetings at Grand Pacific, 8 P. M.

CLINICS. *Every Monday.*

At Eye and Ear Infirmary, (Peoria and Adams Sts.) 2 P. M.—Prof. Holmes.

At Chicago College, 2 P. M. *Gynecological*—Prof. Merriman.

At Central Dispensary (239 W. Van Buren St.), 2 P.M., *Gynecological*—Dr. Adolphus.

At Mercy Hospital, 2 P. M. *Medical*—Prof. Johnson.

LECTURES. *Every Monday.* At Rush College (18th and Arnold Sts.), 8½ to 12½ o'clock—Profs. Gunn, Miller, Freer and Powell; 4 to 6—Profs. Lyman and Etheridge. At Chicago College, 8½ to 12½—Profs. Jewell, Isham, Hyde and Bond; 3 to 6—Profs. Davis and Nelson, Andrews and Quine, and Roler. At Womans' Hospital College, 9 to 12—Profs. Thompson, Bogue and Blake; 3 to 6—Profs. Paoli, Stevenson and MacDonald.

### TUESDAYS.

SOCIETIES.

*Tuesday, Dec.* 14th — Academy of Sciences, regular meeting, 8 P. M. (263 Wabash Av).

CLINICS. *Every Tuesday.*

At County Hospital, 2 P. M., *Medical*—Prof. Ross; 3 P. M., *Surgical*—Prof. Powell.

At Chicago College, 2 P. M., *Gynecological*—Prof. Roler.

At Mercy Hospital, 2 P. M., *Medical*—Prof. Hollister.

LECTURES. *Every Tuesday.*

At Rush College, 8½ to 12½—Profs. Gunn, Miller, Allen and Parkes; 4—Prof. Lyman. At Chicago College, 8½ to 12½—Profs. Jewell, Isham, Merriman and Bond; 3 to 6—Profs. Davis and Nelson, Andrews and Hatfield, and Byford. At Woman's Hosp. Coll., 9 to 12—Profs. Marguerat, Bartlett and Fitch;—3 to 6, Profs. Delafontaine, Curtis and MacDonald.

### WEDNESDAYS.

CLINICS. *Every Wednesday.*

At County Hospital, 2 P. M., *Ophthalmological*—Dr. Montgomery; 3 P. M., *Gynecological*—Prof. Fitch.

At Chicago College, 2 P. M., *Gynecological*—Prof. Nelson.

At Mercy Hospital, 2 P. M., *Surgical*—Prof. Andrews.

LECTURES. *Every Wednesday.*

At Rush College, 8½ to 12½—Profs. Hay, Freer, Allen and Parkes; 4 to 6—Profs. Lyman and Etheridge. At Chicago College, 8½ to 12½—Profs. Hollister, Isham, Hyde and Bond; 3 to 6—Profs. Davis and Curtis, Jones and Quine, and Roler. At Woman's Hosp. Coll., 9 to 12—Profs. Dyas, Earle and Thompson; 3 to 5—Profs. Paoli and Stevenson.

### THURSDAYS.

CLINICS. *Every Thursday.*

At Eye and Ear Infirmary, 2 P. M.—Prof. Holmes.

At Chicago College, 2 P. M., *Gynecological*—Prof. Merriman.

At Central Dispensary, 2 P. M., *Gynecological*—Dr. Adolphus.

At Mercy Hospital, 2 P. M., *Medical*—Prof. Johnson.

LECTURES. *Every Thursday.*

At Rush College, 8½ to 12½—Profs. Gunn, Miller, Allen and Parkes; 4 to 6—Profs. Ross and Lyman. At Chicago College, 8½ to 12½—Profs. Hollister, Isham, Merriman and Bond; 3 to 6—Profs. Davis and Nelson, Andrews and Hatfield, and Byford. At Woman's Hosp. Coll., 9 to 12—Profs. Marguerat, Bogue and Fitch; 3 to 6—Profs. Delafontaine, Curtis and MacDonald.

### FRIDAYS.

CLINICS. *Every Friday.*

At County Hospital, 2 P. M., *Medical*—Prof. Ross; 3 P. M., *Surgical*—Prof. Powell.

At Chicago College, 2 P. M., *Gynecological*—Prof. Roler.

At Mercy Hospital, 2 P. M., *On Dis. Eye and Ear*—Prof. Jones.

LECTURES. *Every Friday.*

At Rush College, 8½ to 12½—Profs. Gunn, Freer, Allen and Parkes; 4 to 6—Profs. Holmes and Etheridge. At Chicago College, 8½ to 12½—Profs. Hollister, Quine, Hyde and Bond; 3 to 6—Profs. Jones and Curtis, Andrews and Quine, and Roler. At Woman's Hosp. Coll., 9 to 12—Profs. Hotz, Bartlett and Blake; 3 to 5—Profs. Paoli and Stevenson.

### SATURDAYS.

CLINICS. *Every Saturday.*

At Rush College, 2 P. M., *Surgical*—Prof. Gunn; 3 P. M., *Diseases of the Brain and Nervous System*—Dr. Hay.

At Chicago College, 2 P. M., *Gynecological*—Prof. Nelson; *Surgical*—Prof. Andrews; 3 P. M., *Medical*—Prof. Davis.

LECTURES. *Every Saturday.*

At Rush College, 8½ to 12½—Profs. Hay, Miller, Freer and Parkes. At Chicago College, 8½ to 12½—Profs. Hollister, Quine, Sherman and Hatfield; 3 to 6—Profs. Davis and Nelson, Andrews and Quine, and Byford. At Woman's Hosp. College, 9 to 12—Profs. Marguerat, Earle and Fitch; 4 to 6—Profs. Curtis and MacDonald; 3 to 5—Prof. Thompson.

☞ There are six special Clinics daily at the South Side Dispensary (Mercy Hospital) for students of the Chicago College. A daily Clinic is given at Dispensary of Hospital for Women and Children, 1½ P. M., for students of Woman's Hospital College.

